# Supplementary material for: Surface Properties of Colloidal Particles Affect Colloidal Self-Assembly in Evaporating Self-Lubricating Ternary Droplets
Source: ACS Appl Mater Interfaces. 2021 Dec 21;14(1):2275–90. doi: 10.1021/acsami.1c19241 (PMC8763378; doi:10.1021/acsami.1c19241)
Supplement: Supplementary file 1 — am1c19241_si_001.pdf [file am1c19241_si_001.pdf]

Supporting Information to

Surface properties of colloidal particles affect colloidal self-assembly in evaporating self-lubricating ternary droplets

Olga Koshkina, *,†,# Lijun Thayyil Raju,‡,# Anke Kaltbeitzel,† Andreas Riedinger,† Detlef Lohse,‡,¶ Xuehua Zhang,*,§,‡ Katharina Landfester*,†

†Max Planck Institute for Polymer Research, 55128 Mainz, Germany

‡Physics of Fluids Group, Max Planck Center for Complex Fluid Dynamics, MESA+ Institute and J. M. Burgers Center for Fluid Dynamics, University of Twente, 7500 AE, Enschede, The Netherlands

¶Max Planck Institute for Dynamics and Self-Organisation, 37077 Göttingen, Germany

§Department of Chemical and Materials Engineering, University of Alberta, Edmonton T6G1H9 Alberta, Canada

# These authors contributed equally to the work

E-mail:

o.koshkina@utwente.nl, xuehua.zhang@ualberta.ca, landfester@mpip-mainz.mpg.de

Table of content

S1. Adhesion energy of particles at liquid-liquid interfaces S-3

S2. Final deposits and their characterization S-4

S3. Additional data on the evaporation process S-7

S4. Additional characterization of the static Ouzo mixtures S-10

S4.1 Size and Intensity S-10

S4.2 Stability of the Static Ouzo Mixtures S-13

S5. Merging of outer-shell of dehydrated unmodified silica particles S-16

S6. Formation of film of particles in droplets containing amine-coated silica particles S-17

S7. Accumulation of the silica particles and the Pickering microdroplets at different interfaces S-18

S7.1 Sedimentation versus drop interface motion S-19

S7.2 Stokes number S-20

S7.3 Peclet Number S-21

S7.4 Conclusion S-23

S8. Internal structure of the supraparticles and the flat deposits S-24

S9. The arrangement of the colloidal particles at the surface of the deposit S-26

S10. References S-27

S1. Adhesion energy of particles at liquid-liquid interfaces

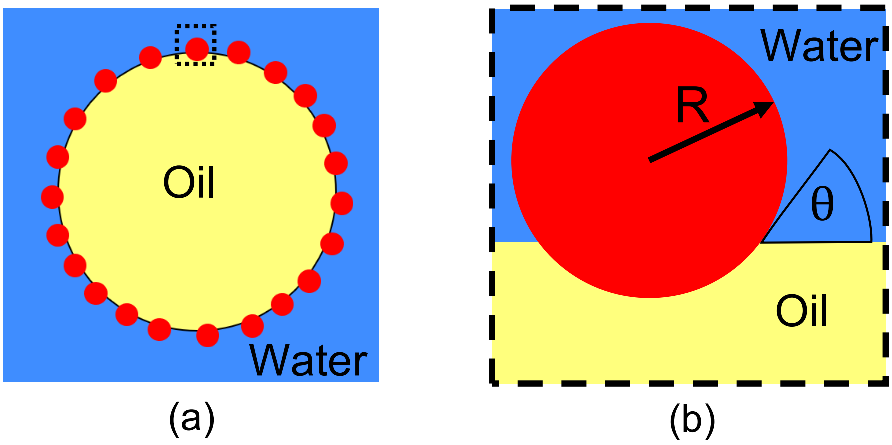

Figure S1. (a) Schematic of colloidal particles adsorbed at the oil-water interface of an oil droplet in water. (b) Zoomed in schematic of a single particle, showing the contact angle θ.

Consider a colloidal particle sitting at oil-water interface with contact angle θ as shown in Figure S1b. Neglecting the curvature of the oil-water interface, the free energy of adsorption (ΔF_{ads}) of the particle is given by [1-3]

$\Delta F_{ads} = -\pi R^2 \gamma_{o-w}(1 - \cos\theta)^2$, for $\theta < 90°$,

$\Delta F_{ads} = -\pi R^2 \gamma_{o-w}(1 + \cos\theta)^2$, for $\theta > 90°$,

where R is the radius of the particle and γ_{o-w} is the surface tension of the oil-water interface. The free energy of adsorption is minimum when $\theta = 90°$. To remove the particles from the oil-water interface, one needs to supply $-\Delta F_{ads}$ amount of energy. Thus, the adsorption of particles is strongest and the emulsions have highest stability when $\theta = 90°$ (when $-\Delta F_{ads}$ is maximum).[2]

S2. Final deposits and their characterization

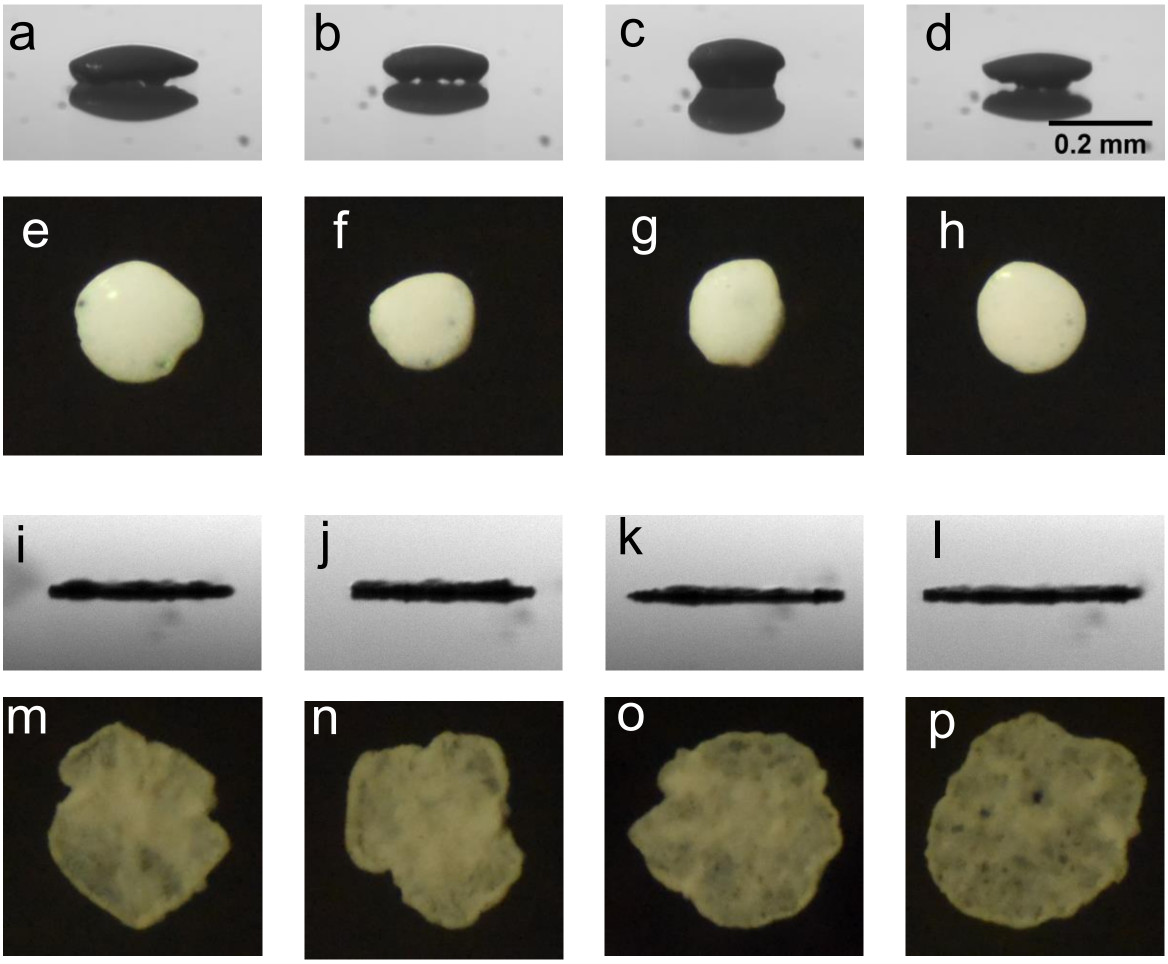

Figure S2. Side view shadowgraph (a-d and i-l) and top view (e-h and m-p) images of the final deposit obtained after evaporation of Ouzo droplets that were loaded with hydrated (a-h) or dehydrated (i-p) unmodified silica particles. Scale bar 0.2 mm.

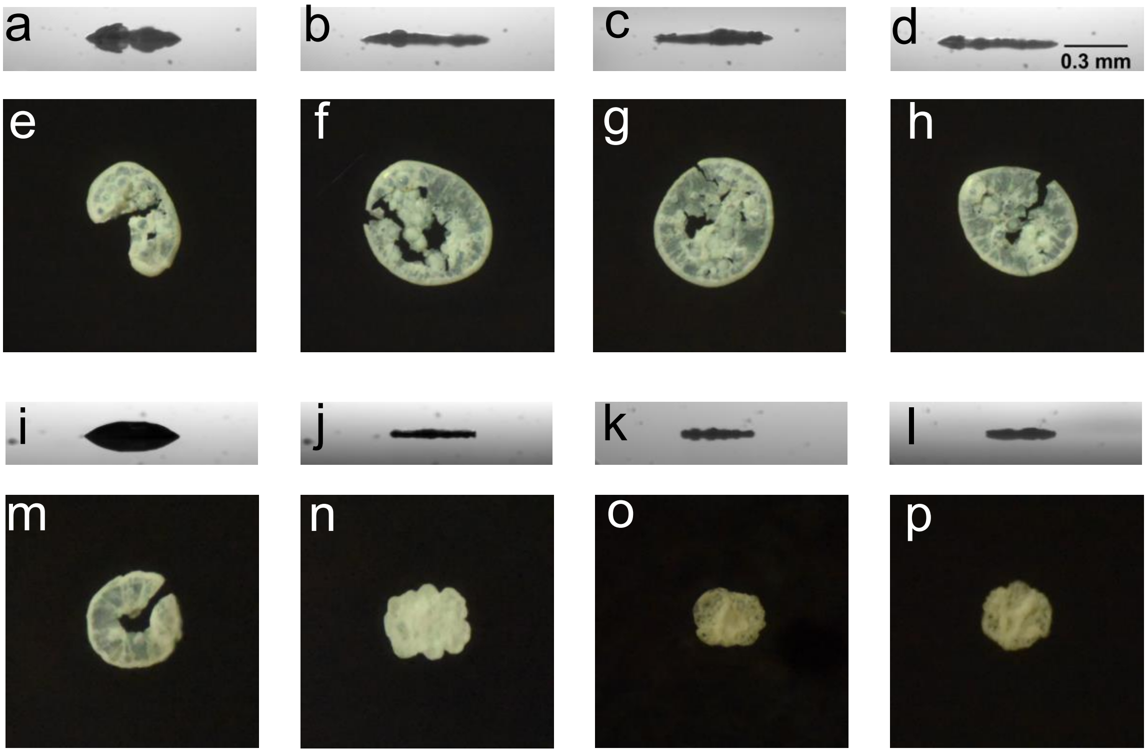

Figure S3. Side view shadowgraph (a-d and i-l) and top view (e-h and m-p) images of the final deposit obtained after evaporation of Ouzo droplets that were loaded with hydrated (a-h) or dehydrated (i-p) amine-coated silica particles. We note that the deposit in (i) might seem like a supraparticle in side view. But the corresponding top view image in (m) clearly shows the strong similarity with the other flat film deposits. Moreover, the lateral width (quantified by area equivalent diameter based on top view image) is also 0.43 mm, which is much larger than the area equivalent diameter of the supraparticles (~0.21 mm for supraparticles of hydrated silica particles; see Figure S5), confirming that a flat film is formed. Scale bar 0.3 mm.

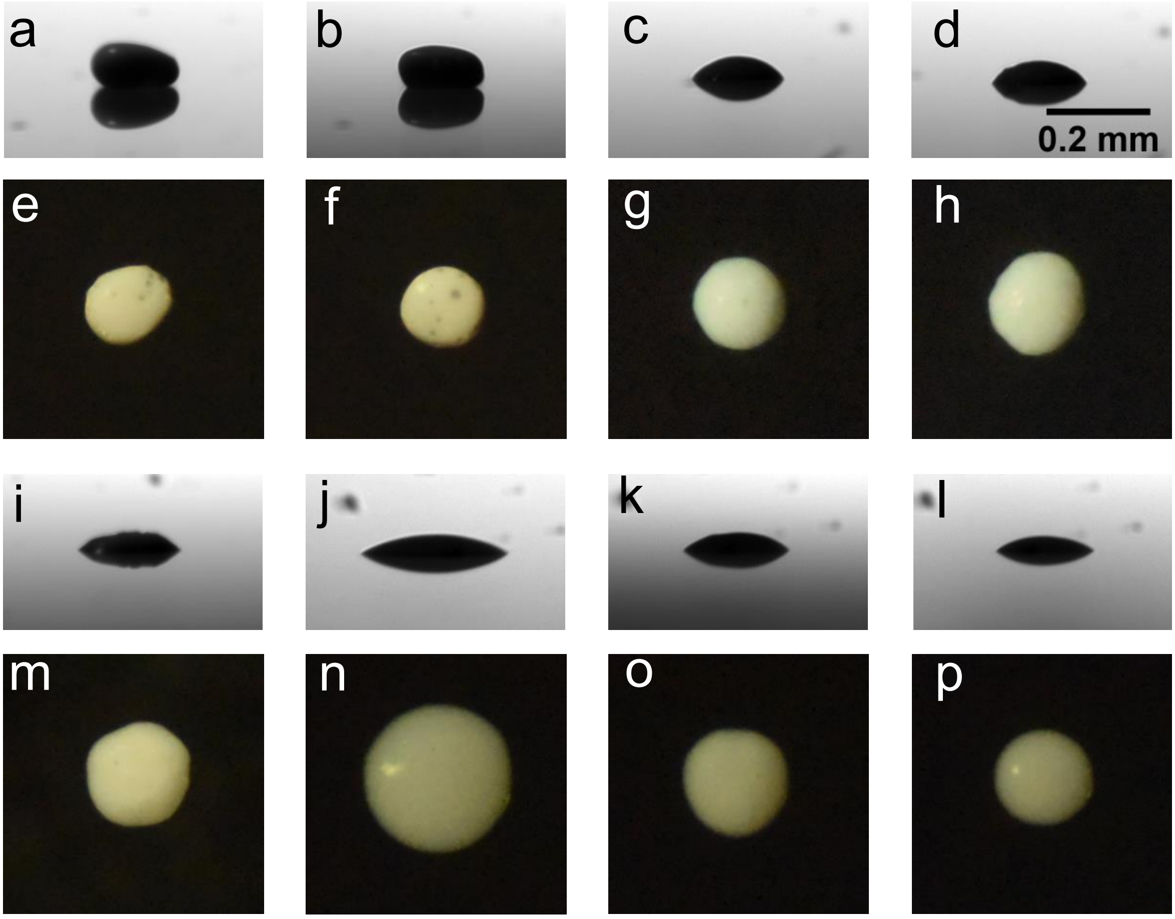

Figure S4. Side view shadowgraph (a-d and i-l) and top view (e-h and m-p) images of the final deposit obtained after evaporation of Ouzo droplets that were loaded with hydrated (a-h) or dehydrated (i-p) PEGylated silica particles. Scale bar 0.2 mm.

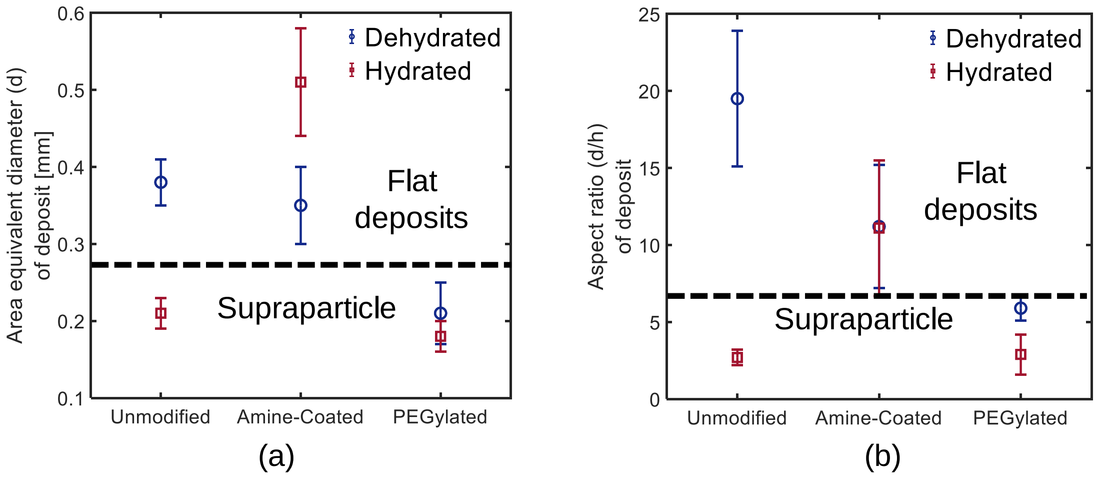

Figure S5. Characterization of final deposits obtained after evaporation of colloidal Ouzo droplets containing different kinds of silica particles. The final deposits are characterized by their respective (a) area equivalent diameter (when viewed from top view) and (b) aspect ratio (defined as d/h, where d is the area equivalent diameter and h is the maximum height of the final deposit). (a) and (b) quantify the difference in shapes of the supraparticles and the flat deposits.

S3. Additional data on the evaporation process

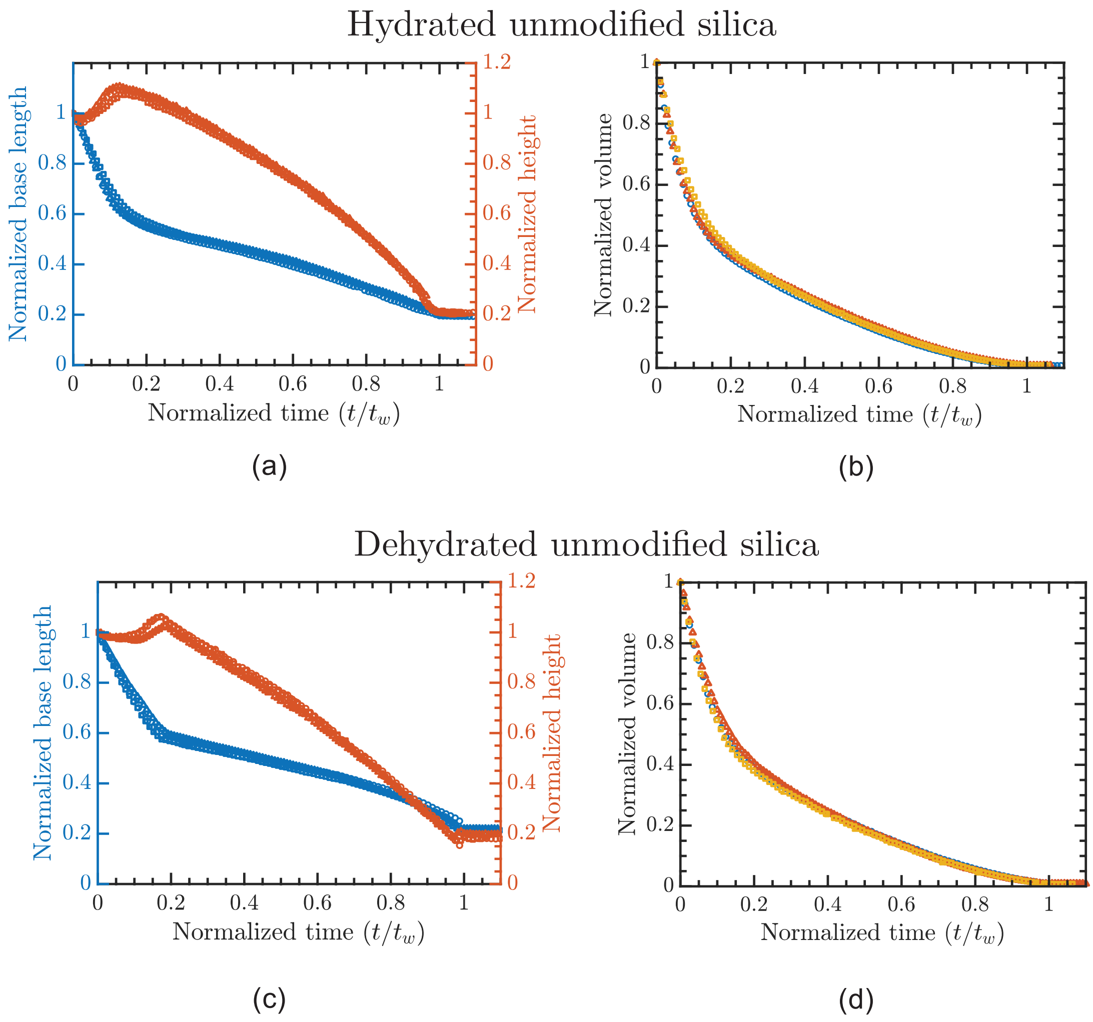

Figure S6. Plots of normalized base length and height (a, c), and volume (b, d) with normalized time for hydrated unmodified silica (a and b) and dehydrated unmodified silica (c and d).

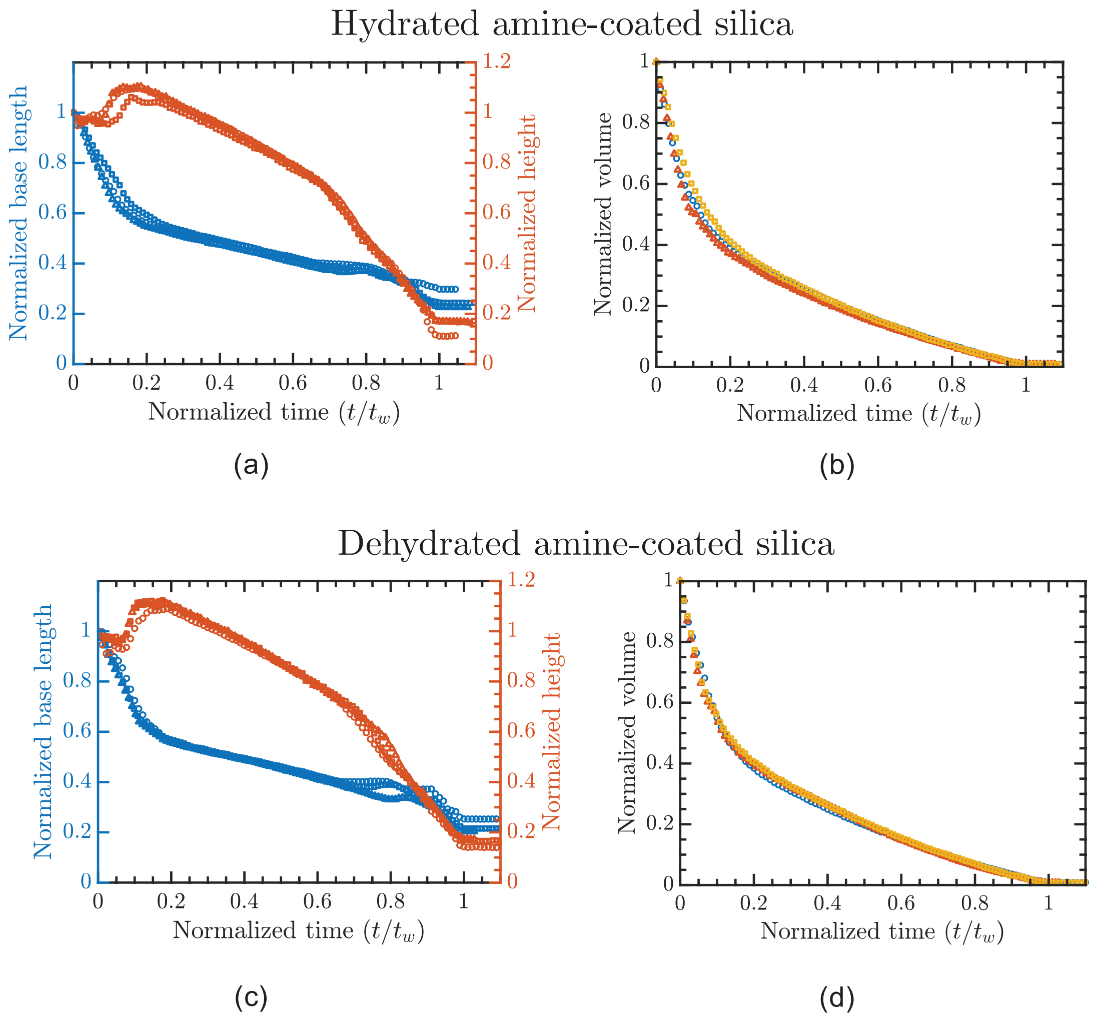

Figure S7. Plots of normalized base length and height (a, c), and volume (b, d) with normalized time for hydrated amine-coated silica (a and b) and dehydrated amine-coated silica (c and d).

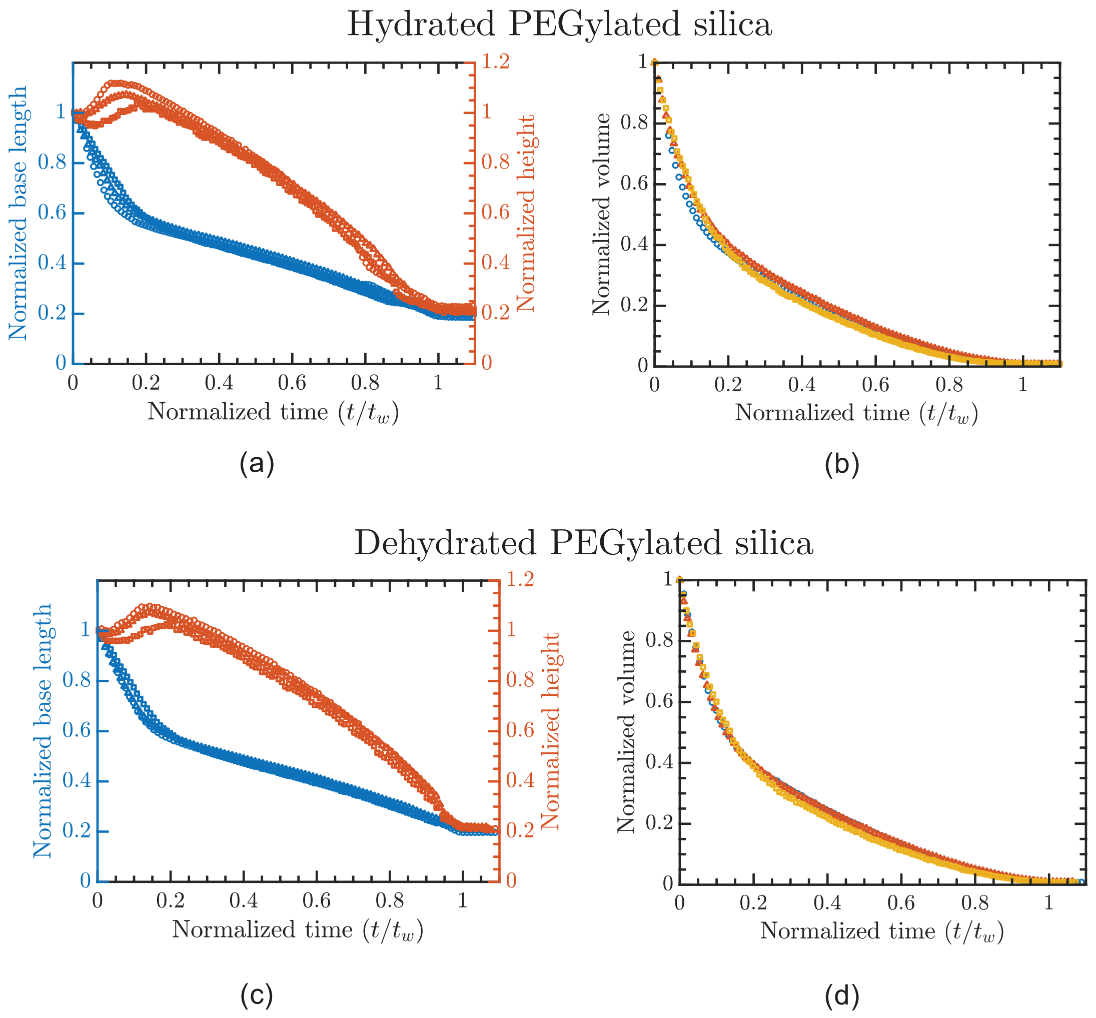

Figure S8. Plots of normalized base length and height (a, c), and volume (b, d) with normalized time for hydrated PEGylated silica (a and b) and dehydrated PEGylated silica (c and d).

S4. Additional characterization of the static Ouzo mixtures

S4.1 Size and Intensity

Figure S9 shows additional zoomed-in images obtained from fluorescence confocal microscopy, showing that surface modifications affect particle-oil interactions. The images are overlay of fluorescence signals from the particles (red) and oil (yellow). It further confirms that hydrated silica particles do not interact with oil droplets while dehydration and the surface modification with amine and PEG, lead to the adsorption of the particles onto the surface of oil droplets.

Figure S10 shows the differences in the size-distribution of the phase separated oil-droplets for each case and Figure S11 shows the volume weighted mean radius (R_{oil}) of these oil microdroplets, as imaged using confocal microscopy. In the Ouzo mixtures with hydrated unmodified particles, the phase separated oil-droplets showed a broad size distribution that was shifted towards larger sizes ($r_{oil,\ hydrated\ unmodified}$ = 23±8 μm), compared to the Ouzo mixture with dehydrated unmodified particles ($r_{oil,\ dehydrated\ unmodified}$ = 6±3 μm, Figure S10). For amine-coated and PEGylated particles, the differences in droplet sizes between dried and hydrated particles were less pronounced, as plotted in Figure S11. Note, however, that the analysis of droplets size is challenging, as the droplets move and sediment; additionally, the typical polydispersity of droplets at the spinodal region make the further analysis difficult.

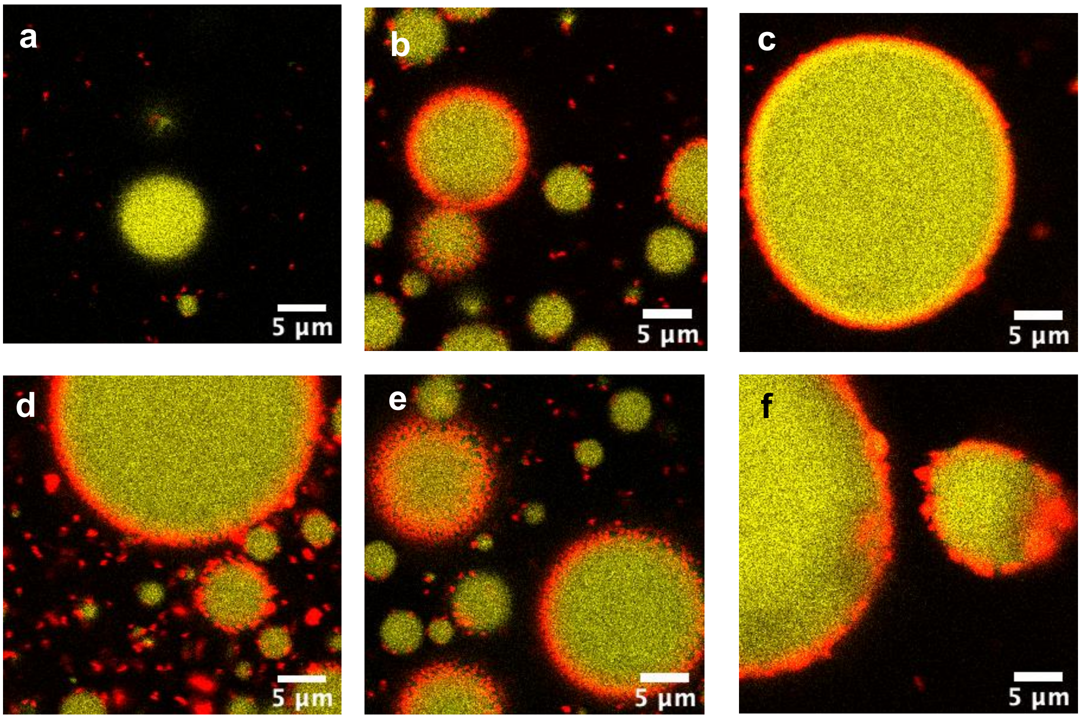

Figure S9. Additional images of colloidal Ouzo mixtures studied by confocal microscopy under non-evaporating conditions. (a-c) hydrated and (d-f) dehydrated silica particles; (a, d) non-modified silica particles, (b,e) PEGylated silica particles, (c,f) amine-coated silica particles. As the amine-coated silica particles tended to accumulate at the lower cover slip, they are not visible in the dispersed medium in the current image. Silica in red (rhodamine) and oil in yellow (perylene). All images are taken at similar settings; intensities are comparable. Scale bar 5 µm.

Table S1. Determination of size of oil microdroplets in the non-evaporating Ouzo mixtures that contain particles with different surface modification (R represents radius of oil microdroplets averaged by number).

Particles used in the mixture	R / µm	R_{max} /µm	Intensity shell, all droplets	Intensity shell, R > 10 µm
Hydrated unmodifed[i]	7±5	32	8	6
Dried unmodified	4±1	14	88	108
Hydrated amine-coated	4±2	21	71	70
Dried amine-coated	6±4	21	54	55
Hydrated PEGylated	4±2	22	52	55
Dried PEGylated	4±1	10	46	41

[i] This Ouzo-mixture showed a phase separation visible by naked eye after standing for about 30 min. Thus, since this mixture was undergoing phase separation, the size determination is not precise.

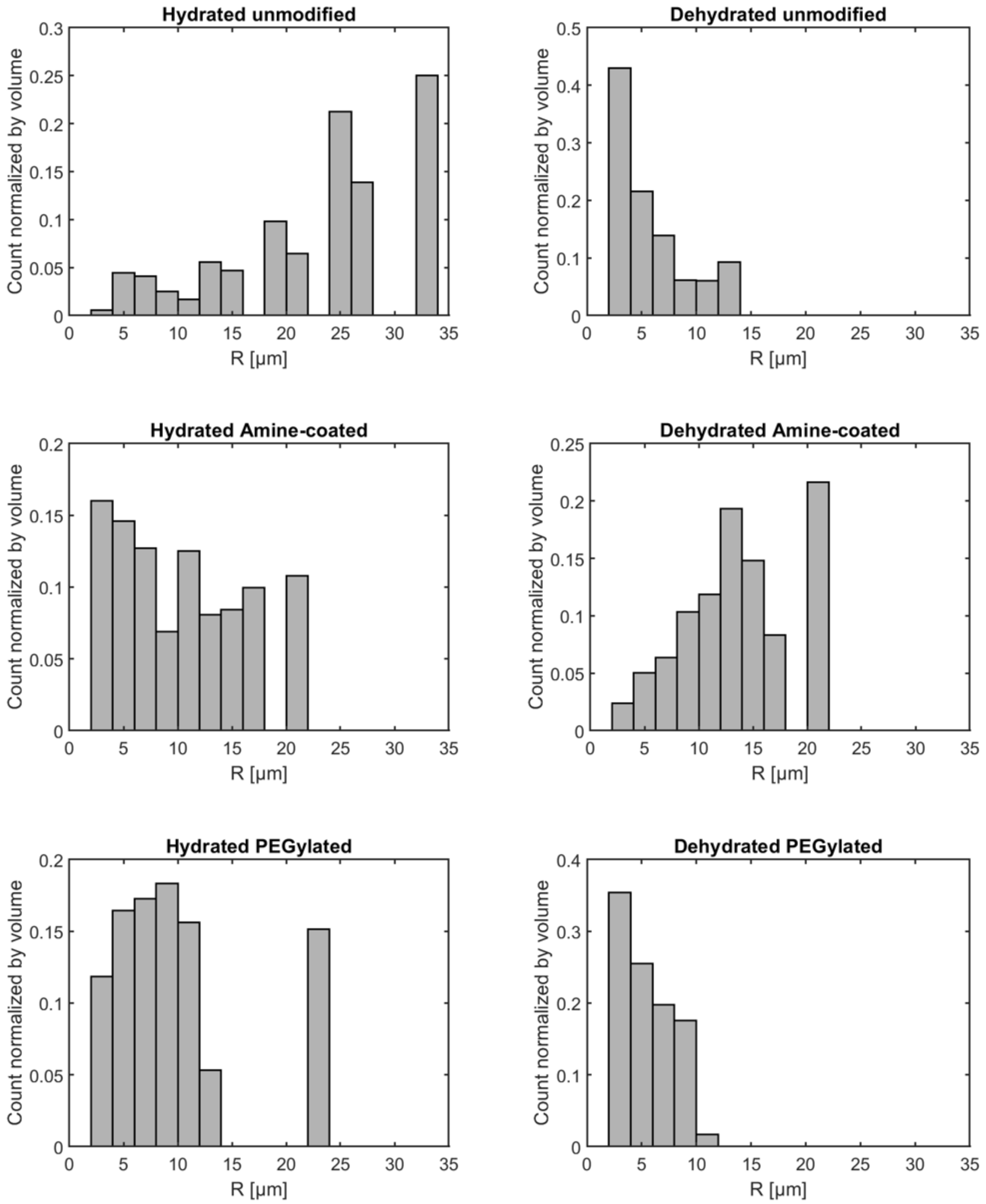

Figure S10. Volume weighted distribution of radius of the oil microdroplets obtained in the non-evaporating Ouzo-mixtures that contain silica particles with different surface modifications.

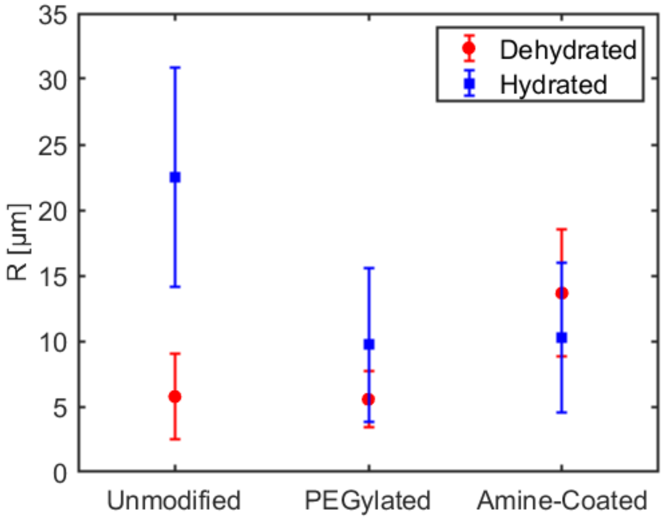

Figure S11. Average radius (averaged by volume) of the oil microdroplets obtained in the non-evaporating Ouzo-mixtures that contain silica particles with different surface modifications.

S4.2 Stability of the Static Ouzo Mixtures

As all particles that formed shells appeared stable when observed by naked eye, we additionally monitored their stability by the confocal microscopy (Figure S12-Figure S14). The experiments in this section were performed independently on different days from the experiments used for Figure S10 and Figure S11, additionally showing the reproducibility of the shell formation. The mixture with non-modified hydrated silica particles was not included, as it underwent a phase separation after approximately 30 min that was visible by a naked eye, as expected for an Ouzo-mixture close to the spinodal line. Confocal microscopy confirmed that the Pickering-like droplets remained stable for at least one week. The analysis of droplet sizes showed a shift of the size distribution towards larger, as a result of aging.

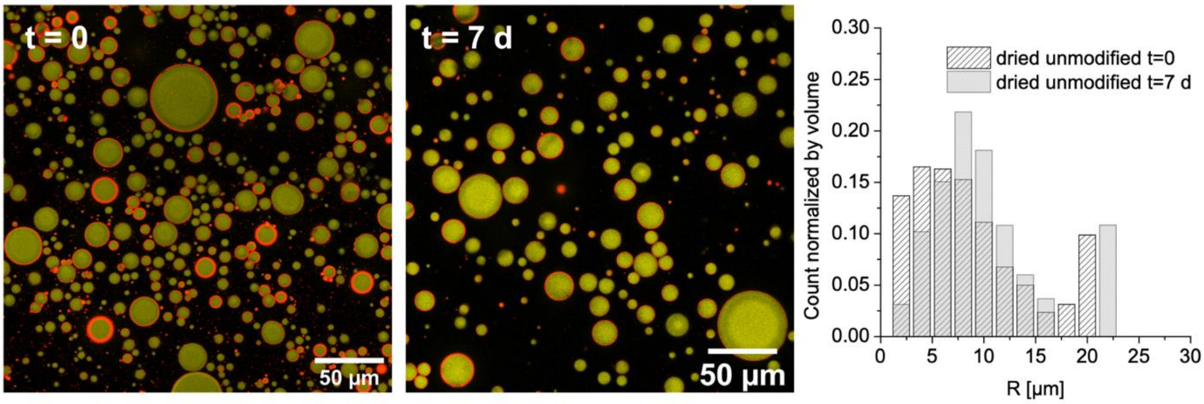

Figure S12. Stability of the colloidal Ouzo mixtures that contain dried unmodified silica particles. Colloidal Ouzo mixture after preparation (left), after one week (middle), and the volume averaged size distributions are shown. Silica in red (rhodamine) and oil in yellow (perylene). Scale bar 50 µm.

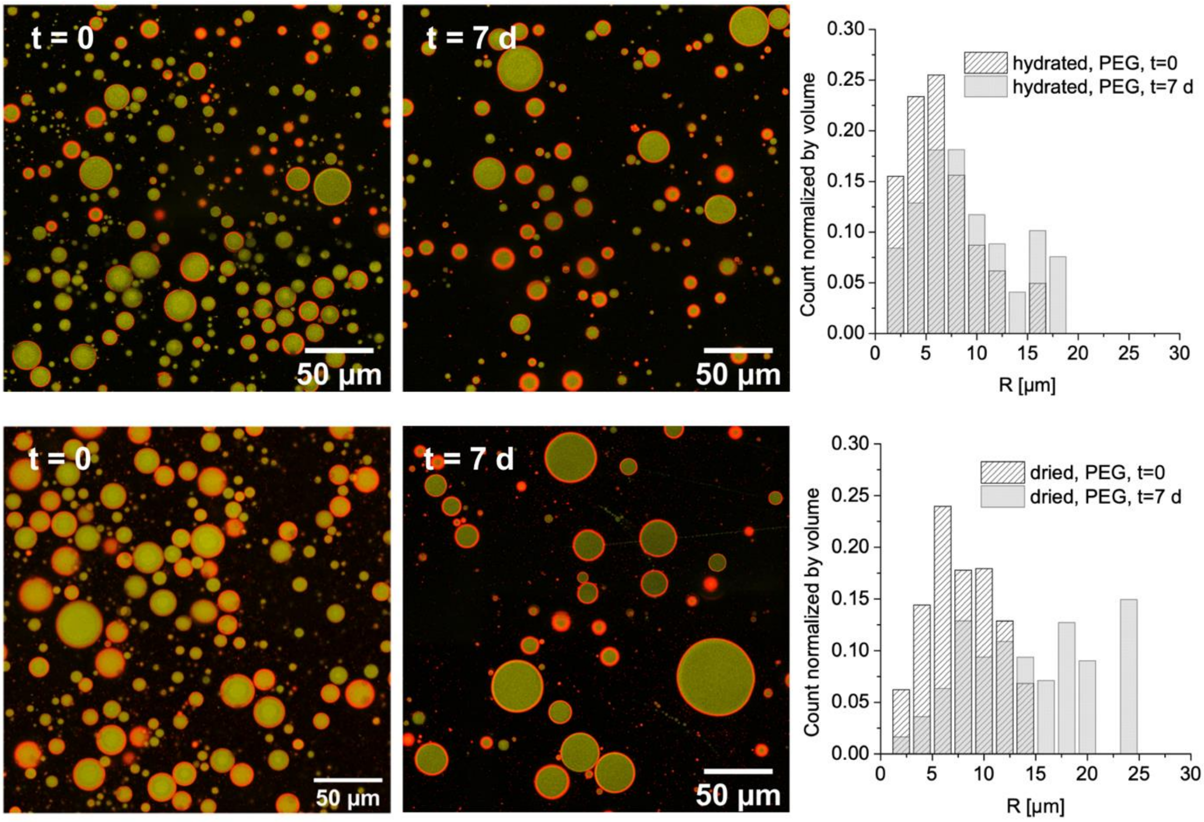

Figure S13. Stability of the colloidal Ouzo mixtures that contain PEGylated silica particles. Upper row: hydrated particles; lower row: dried particles. Colloidal Ouzo mixture after preparation (left), after one week (middle), and the volume averaged size distributions are shown. Silica in red (rhodamine) and oil in yellow (perylene). Scale bar 50 µm.

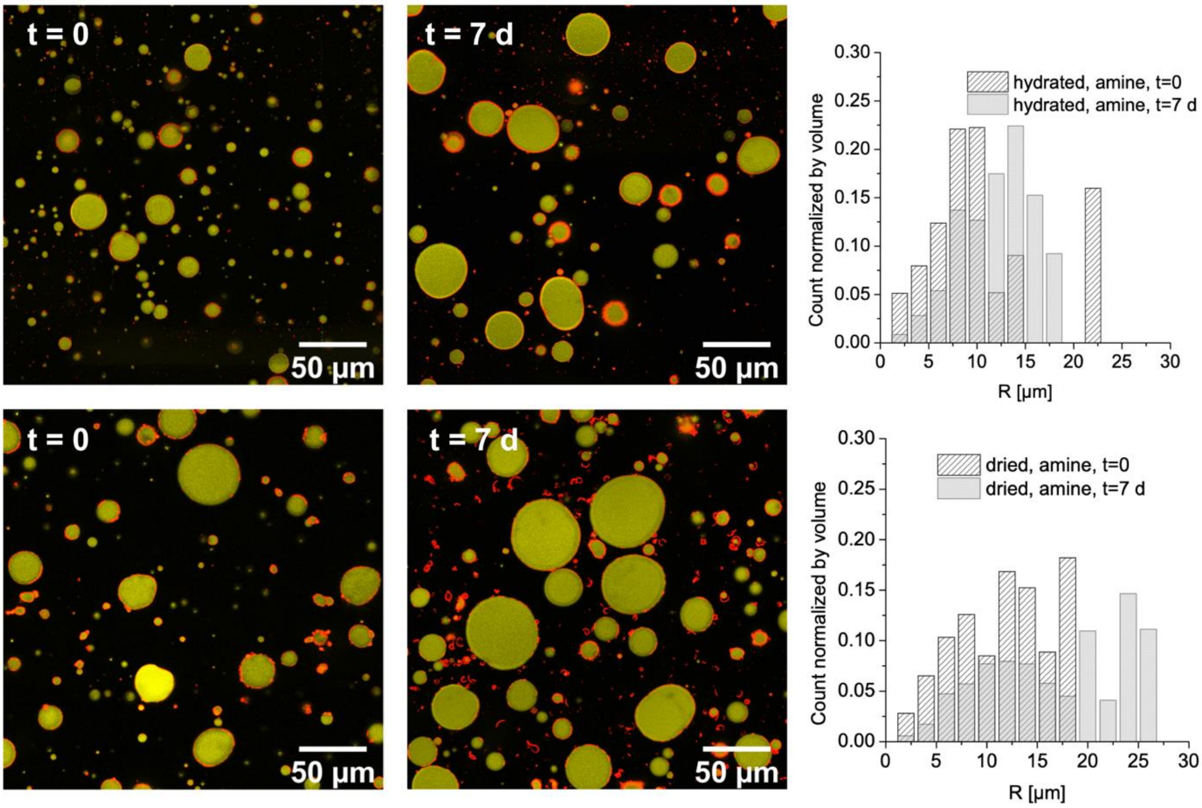

Figure S14. Stability of the colloidal Ouzo mixtures that contain amine-coated silica particles. Upper row: hydrated particles; lower row: dried particles. Colloidal Ouzo mixture after preparation (left), after one week (middle), and the volume averaged size distributions are shown. Silica in red (rhodamine) and oil in yellow (perylene). Scale bar 50 µm.

S5. Merging of outer-shell of dehydrated unmodified silica particles

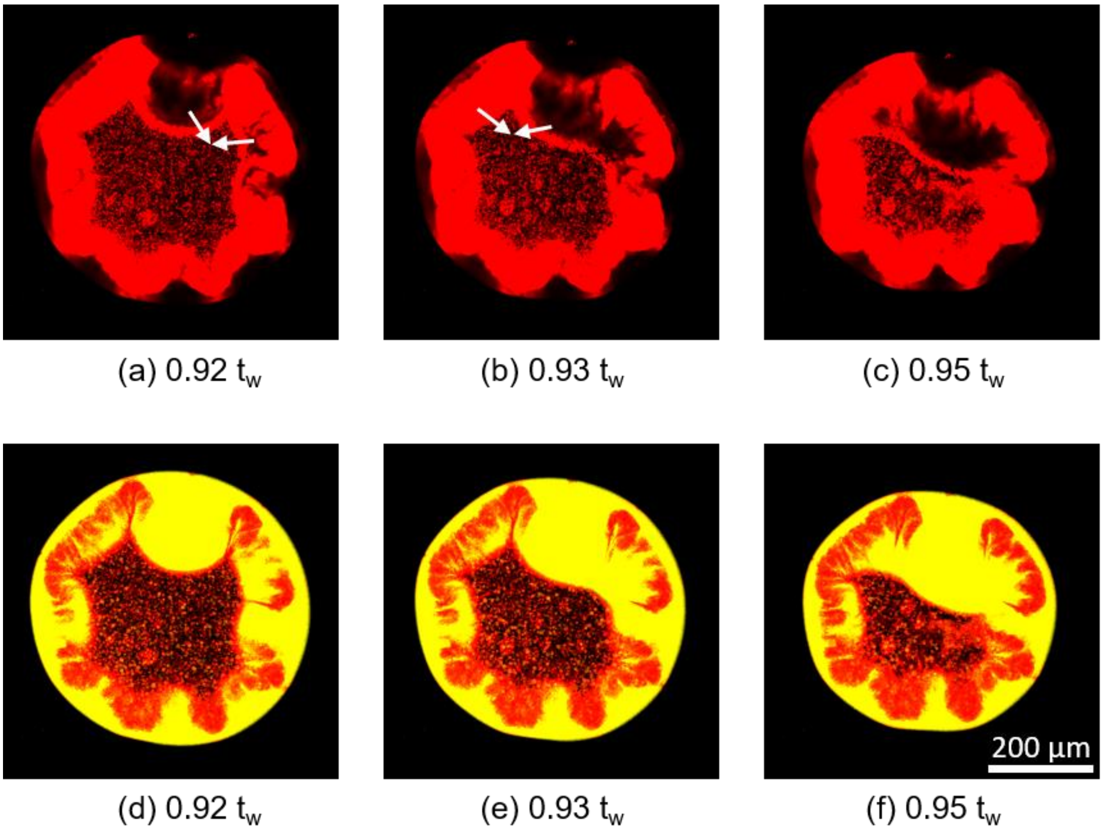

Figure S15. Merging of the asymmetric petal-like sides of the outer shell observed in an evaporating Ouzo droplet containing dehydrated unmodified silica particles. Silica in red (rhodamine) and oil in yellow (perylene). (a) – (c) show only the spatial distribution of silica particles, while (d) - (f) additionally show the presence of oil at the same time as in (a) – (c). The white arrows show the location and direction in which the merging occurs. Scale bar 200 µm.

S6. Formation of film of particles in droplets containing amine-coated silica particles

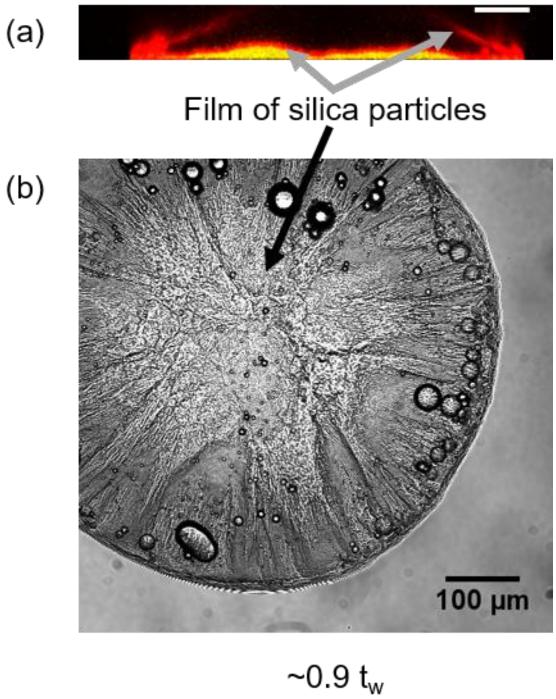

Figure S16. Confocal microscopy shows the formation of film of particles in the droplets with amine-modified silica particles. (a) vertical cross section, reconstructed from layer-wise scanning. Silica in red (rhodamine) and oil in yellow (perylene). (b) Image of a horizontal plane, viewed in transmission channel. The images in (a) and (b) are of two different droplets. Scale bar 100 μm.

S7. Accumulation of the silica particles and the Pickering microdroplets at different interfaces

The accumulation of the silica particles and the Pickering microdroplets at the various interfaces of the droplet could be influenced by processes such as advection by the flow inside the Ouzo droplet, Brownian diffusion, sedimentation, and capture by the moving interfaces of the Ouzo droplet. To analyze the different time scales associated with these processes, we consider a particle-coated oil droplet, *i.e. a Pickering microdroplet*, having radius R (such that $R = r_{oil} + d_{particle}$) in a fluid (water) of density ρ_{water} (Figure S17a). Note that the times scales and velocity scales estimated in the following section apply to the time after the intense Marangoni flow in the Ouzo droplet has ceased i.e. to the time when there is no significant amount of ethanol left in the Ouzo droplet (marked by the sharp change in slope of the curve of volume vs time in Figure 4a of the main text).

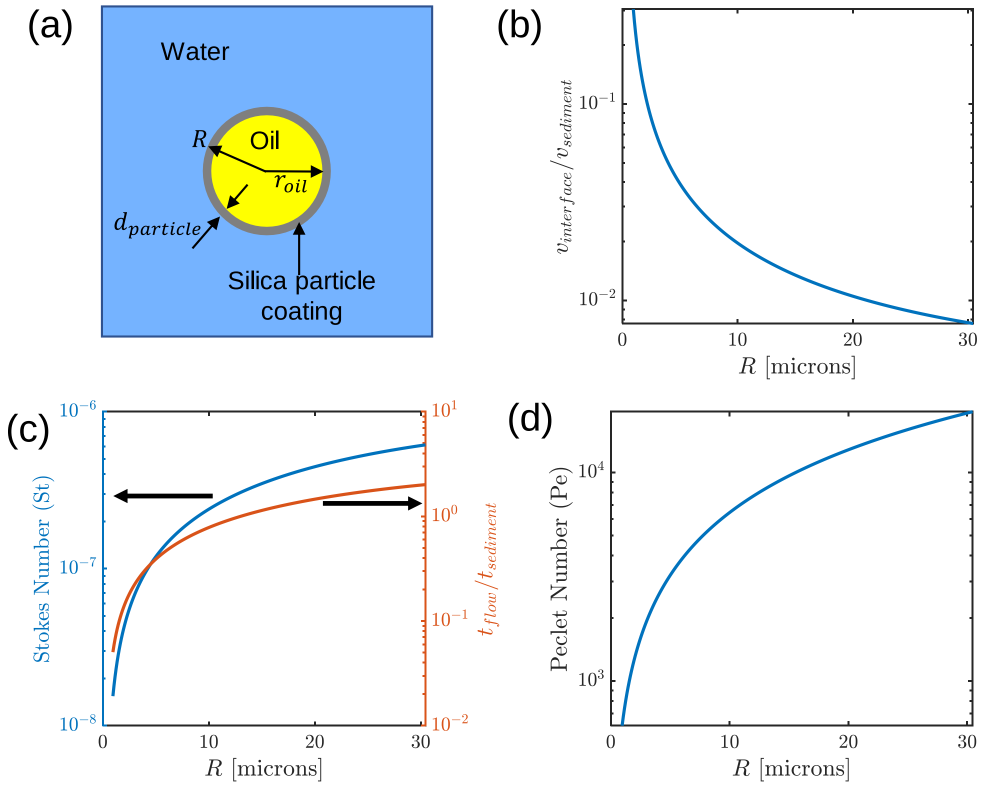

Figure S17. Comparison of the different phenomena that govern the motion of Pickering microdroplets, namely (1) capture of the Pickering microdroplets by the moving interfaces of the Ouzo droplet, (2) sedimentation, (3) flow field in the droplet, and (4) Brownian diffusion. (a) Schematic of a Pickering oil-microdroplet. (b-d) Plots show the variation in (b) the ratio of interface velocity ($v_{interface}$) and sedimentation velocity ($v_{sediment}$), (c) Stokes number (St) and the ratio of characteristic timescale of the flow field (t_{flow}) to characteristic sedimentation time

($t_{sediment}$), and (d) Peclet number (Pe), for different radii ($R = r_{oil} + d_{particle}$) of the Pickering microdroplet.

S7.1 Sedimentation versus drop interface motion

(a) Analysis for Pickering microdroplets

We consider a Pickering microdroplet sedimenting in a still fluid (Figure S17a). Let M_1 be the mass of the Pickering microdroplet such that

$$M_1 \approx \rho_{oil}\left(\frac{4}{3}\pi r_{oil}^3\right) + \rho_{silica}\left(\frac{4}{3}\pi\right)\left(\left(r_{oil} + d_{particle}\right)^3 - r_{oil}^3\right),$$

where we have approximated the mass of the layer of silica particles on the oil drop as the mass of a thin spherical shell of inner radius r_{oil} and outer radius $r_{oil} + d_{particle}$. Let M_2 be the mass of water displaced by the Pickering microdroplet, such that

$$M_2 = \rho_{water}\left(\frac{4}{3}\pi\left(r_{oil} + d_{particle}\right)^3\right)$$

The terminal sedimentation velocity of such a droplet is given by the Stokes velocity

$$v_{sediment,Pickering} = \frac{(M_1 - M_2)g}{6\pi\mu_{water}R}$$

Taking the average height of the droplet as h~0.3 mm, the sedimentation time of the Pickering microdroplet will be

$$t_{sediment,Pickering} = \frac{h}{v_{sediment,Pickering}}$$

To determine whether the Pickering-microdroplets might sediment before getting captured by the interface, we calculate the ratio of the average velocity of the moving interface ($v_{interface\ average}$) to the sedimentation velocity ($v_{sediment,oil}$) of the Pickering oil microdroplets. The average rate of decrease of the height of the droplet is calculated to be $v_{interface}$ = 4.6 x 10^{-4} mm/s using the curves of height versus time obtained from shadowgraph measurements (Figure S6-Figure S8). Figure S17b shows the variation in $\left(\frac{v_{interface}}{v_{sediment}}\right)_{Pickering}$ with the radius, *R,* of the Pickering microdroplet. Thus, $\left(\frac{v_{interface}}{v_{sediment}}\right)_{Pickering}$ ≈ O(0.1) – O(0.01), showing that

sedimentation of Pickering microdroplets is faster than the movement of the air-liquid interface of the droplet.

(b) Analysis for individual particles

For a silica particle, the terminal sedimentation velocity is again given by the Stokes velocity

$$v_{sediment,particle} = \frac{\left(\frac{4}{3}\pi\left(\frac{d_{particle}}{2}\right)^3\right)\left(\rho_{particle} - \rho_{water}\right)g}{6\pi\mu_{water}\left(\frac{d_{particle}}{2}\right)} = \frac{1}{18} * \frac{d_{particle}^2\left(\rho_{particle} - \rho_{water}\right)g}{\mu_{water}},$$

which is the well-known formula for the sedimentation velocity[4]. The density of the particles is $\rho_{particle} = \rho_{silica} \approx 1.8$ g/cm^3. Substituting the values, we get $\left(\frac{v_{interface}}{v_{sediment}}\right)_{particle} \approx 4$. Thus, the interface motion is marginally faster than the sedimentation of individual particles. Finally, using $t_{sediment} = \frac{h}{v_{sediment}}$, we get $t_{sediment,particles} = 3 \times 10^3$ s, which is slightly larger than the evaporation time scale, $t_w = 930 \pm 170$ s, in our system.

S7.2 Stokes number

The motion of the particles and the Pickering microdroplets is also influenced by the fluid velocities in the surrounding medium. Hence, we look at Stokes number. The ability of a sphere of radius *R* to act as a faithful tracer of the flow can be understood using the Stokes number St, defined as[5]

$$St = \frac{t_{response}}{t_{flow}},$$

where $t_{response}$ is the response time of the sphere and t_{flow} is the characteristic velocity of flow in the surrounding medium.

(a) Analysis for Pickering microdroplets

For our study, based on the Maxey-Riley equation[5], the response time of a Pickering microdroplet is given by

$$t_{response} = \frac{M_1 - M_2}{6\pi\mu_{water}R}$$

Here, M_1 is the mass of a Pickering microdroplet and M_2 is the mass of water displaced by the Pickering microdroplet, as defined in the previous section.

Further,

$$t_{flow} = \frac{L_{characteristic}}{u_{flow}}$$

$L_{characteristic}$ and u_{flow} are the characteristic length scale and the characteristic fluid velocity, respectively, of the fluid flow in the surrounding medium. We take the radius of the Ouzo droplet after most of the ethanol has evaporated as $L_{characteristic}$ ≈ 0.5 mm, the relevant length scale of the flow. We expect the flow velocities to be ~1-50 μm/s when the intense Marangoni flow has subsided.[6, 7] We take u_{flow} ~ 50 μm/s. Figure S17c shows the variation in Stokes number (*St*) with the radius (*R*) of Pickering microdroplets. The very low magnitude of the Stokes number (*St*) shows that these oil microdroplets can follow the flow field of the surrounding medium. We also compare t_{flow} and $t_{sediment}$ for the Pickering microdroplets in Figure S17c. Since $\left(\frac{t_{flow}}{t_{sediment}}\right)_{Pickering}$ ≈ O(0.1) - O(1), both the flow field and sedimentation can affect the motion of the Pickering microdroplets.

(b) Analysis for individual particles

For a silica particle, the Stokes number can be calculated similarly by taking

$$t_{response} = \frac{2}{9} \frac{\left(\frac{d_{particle}}{2}\right)^2 (\rho_{silica} - \rho_{water})}{\mu_{water}}.$$

Thus we obtain $St_{particle} = \frac{t_{response}}{t_{flow}}$ = 1 x 10^{-9}. Furthermore, $\left(\frac{t_{flow}}{t_{sediment}}\right)_{particle}$ ≈ O(0.001). Thus, for the individual silica particles, the flow field will have much greater influence on the motion of the particles compared to sedimentation.

S7.3 Peclet Number

We define the Peclet number as

$$Pe = \frac{t_{mass\,diffusion}}{t_{interface\,movement}},$$

where $t_{mass\,diffusion}$ is the time scale associated with diffusion of the Pickering microdroplets/particles and $t_{interface\,movement}$ is the time scale associated with the interface movement. A high Peclet number indicates the possibility of accumulation of the Pickering microdroplets/particles close to the moving interface. We have

$$t_{mass\,diffusion} = \frac{l^2}{D_{mass}},$$

where l is the relevant length scale and D_{mass} is the mass diffusion coefficient, given by the Stokes-Einstein relationship

$$D_{mass} = \frac{kT}{6\pi\mu R}.$$

μ is the viscosity of the surrounding medium, k is the Boltzmann constant, *T* is the temperature in Kelvin and *R* is the radius.

We further determine

$$t_{interface\,movement} = \frac{l}{v_{interface\,average}},$$

where $v_{interface\,average}$ is the average velocity of the moving interface. We take l=0.3mm, the average height of the droplet, and $v_{interface\,average}$ = 4.6 x 10^{-4} mm/s, the average rate of decrease of the height of the droplet obtained from shadowgraph measurements (Figure S6-Figure S8).

(a) Analysis for Pickering microdroplets

Using the above expressions and numerical values, we plot the Peclet number of the Pickering microdroplets for various radii, *R* (Figure S17d). The high Peclet numbers indicate the possibility of the oil microdroplets to accumulate close to a moving interface, when only diffusion and interface motion are considered.

(b) Analysis for individual particles

The Peclet numbers for the silica particles can be calculated similarly, resulting in Pe = 1.4 x 10^2. Thus, the air-water interface moves faster compared to the Brownian diffusion of the silica particles. Thus, silica particles can accumulate close to the moving interfaces.

S7.4 Conclusion

In conclusion, for individual silica particles, the motion of the interface of the evaporating droplet is much faster than the mass-diffusion of the particles and marginally faster than the sedimentation of the particles. As a result, the particles accumulate close to the air-water interface and oil-water interface, forming a region of high silica concentration, termed as "*outer shell*" in the main text (as seen in Figure 7 and Figure 8 in the main text).

This outer shell is clearly visible in all the cases except at the air-water interface in the Ouzo droplet containing dehydrated unmodified silica particles (Figure 8a in the main text). Moreover, with dehydrated unmodified silica particles, the oil ring is thinner and a large amount of Pickering microdroplets are seen in the evaporating droplet (Figure 8c,e-g in the main text). The thin oil ring and the large number of Pickering microdroplets indicate that a large number of particles were used to form Pickering microdroplets. Furthermore, the fact that $\left(\frac{v_{interface}}{v_{sediment}}\right)_{Pickering}$ ≈ O(0.1) – O(0.01) and $\left(\frac{t_{flow}}{t_{sediment}}\right)_{Pickering}$ ≈ O(0.1) - O(1) (Figure S17 b,c) suggests that sedimentation and advection by the fluid flow are more prominent effects for Pickering microdroplets rather than the capture by the moving air-liquid interface. Thus a combined effect of the flow field and the sedimentation of Pickering microdroplets could be responsible for the absence of outer shell at the air-water interface for Ouzo droplets containing dehydrated unmodified silica particles.

S8. Internal structure of the supraparticles and the flat deposits

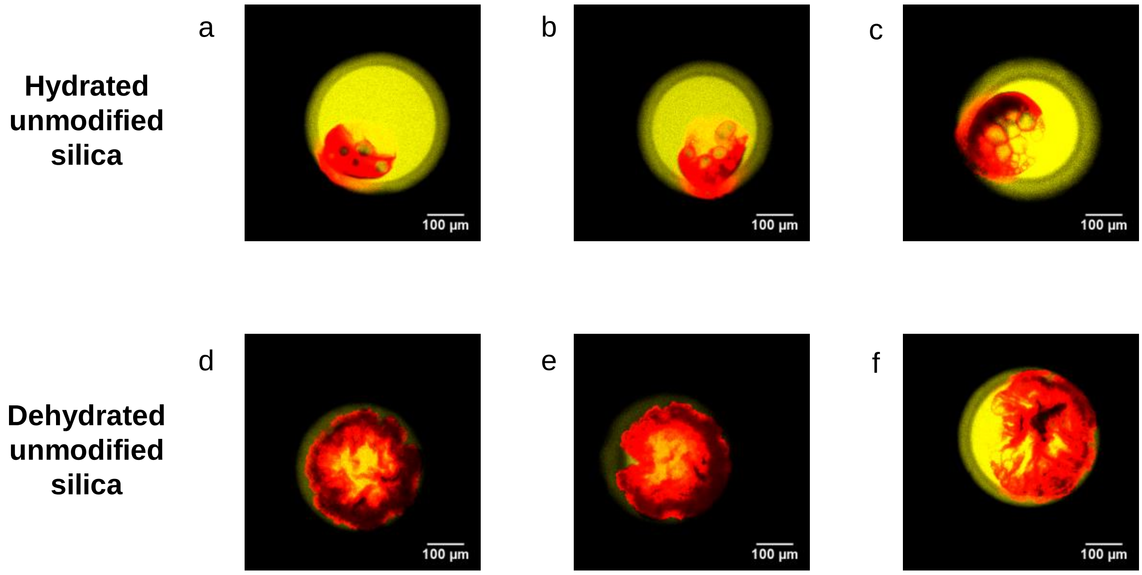

Figure S18. Porosity of the final deposits obtained that were loaded with hydrated (a-c) or dehydrated (d-f) unmodified silica particles. Fluorescence confocal microscopy image showing the distribution of pores inside the assembled final deposit (red), at time $t>t_w$. Oil is shown in yellow. The imaging is done at a plane approximately at mid-height of the deposit. Scale bar 100 µm.

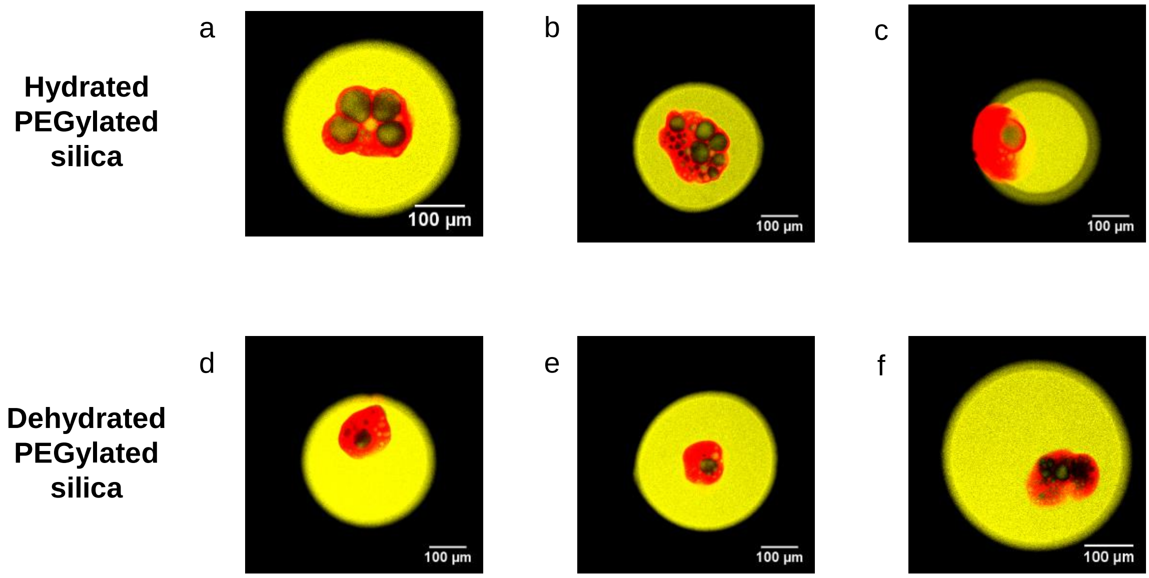

Figure S19. Porosity of the final deposits obtained that were loaded with hydrated (a-c) or dehydrated (d-f) PEGylated silica particles. Fluorescence confocal microscopy image showing the distribution of pores inside the assembled final deposit (red), at time $t>t_w$. Oil is shown in yellow. The imaging is done at a plane approximately at mid-height of the deposit. Scale bar 100 µm.

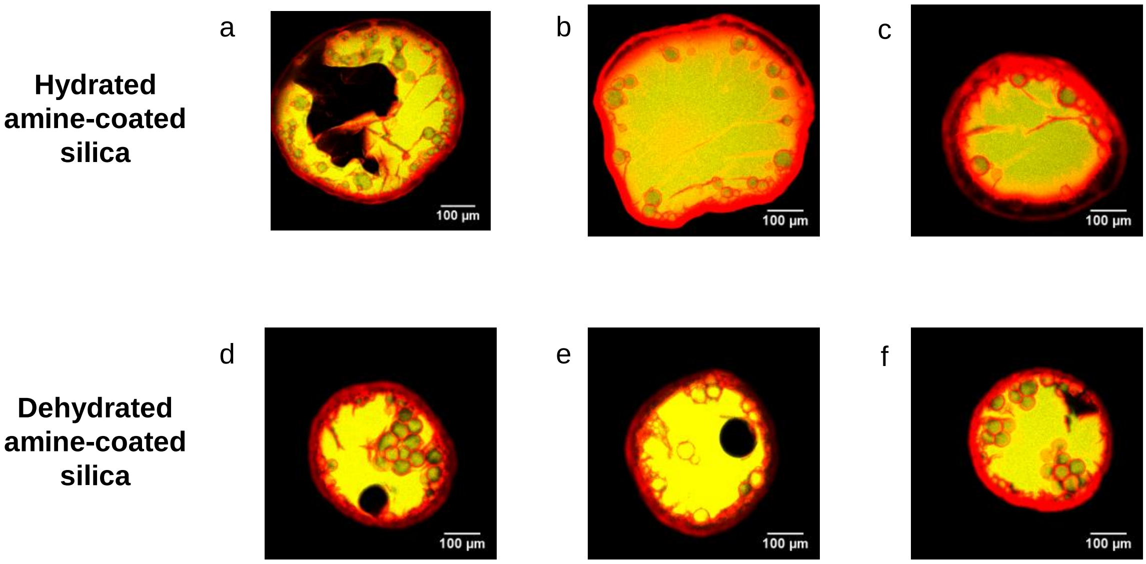

Figure S20. Porosity of the final deposits obtained that were loaded with hydrated (a-c) or dehydrated (d-f) amine-coated silica particles. Fluorescence confocal microscopy image showing the distribution of pores inside the assembled final deposit (red), at time $t>t_w$. Oil is shown in yellow. The imaging is done at a plane approximately at mid-height of the deposit. Scale bar 100 µm.

Previous studies have shown that supraparticles made using self-lubricating droplets have porous internal structure.[8] In the present work, using confocal microscopy, we also show that there are large hollow spaces (pores) in the supraparticles made of hydrated unmodified silica particles (Figure S18 a-c), PEGylated silica particles (Figure S19), and the flat deposit made of amine-coated silica particles (Figure S20). However, the flat deposits made of dehydrated silica particles (Figure S18 d-f) do not show such large pores. These differences in the internal structure could be determined by the size distribution of the Pickering microdroplets in the evaporating droplet. Further studies are required to understand these differences and control the porosity.

S9. The arrangement of the colloidal particles at the surface of the deposit

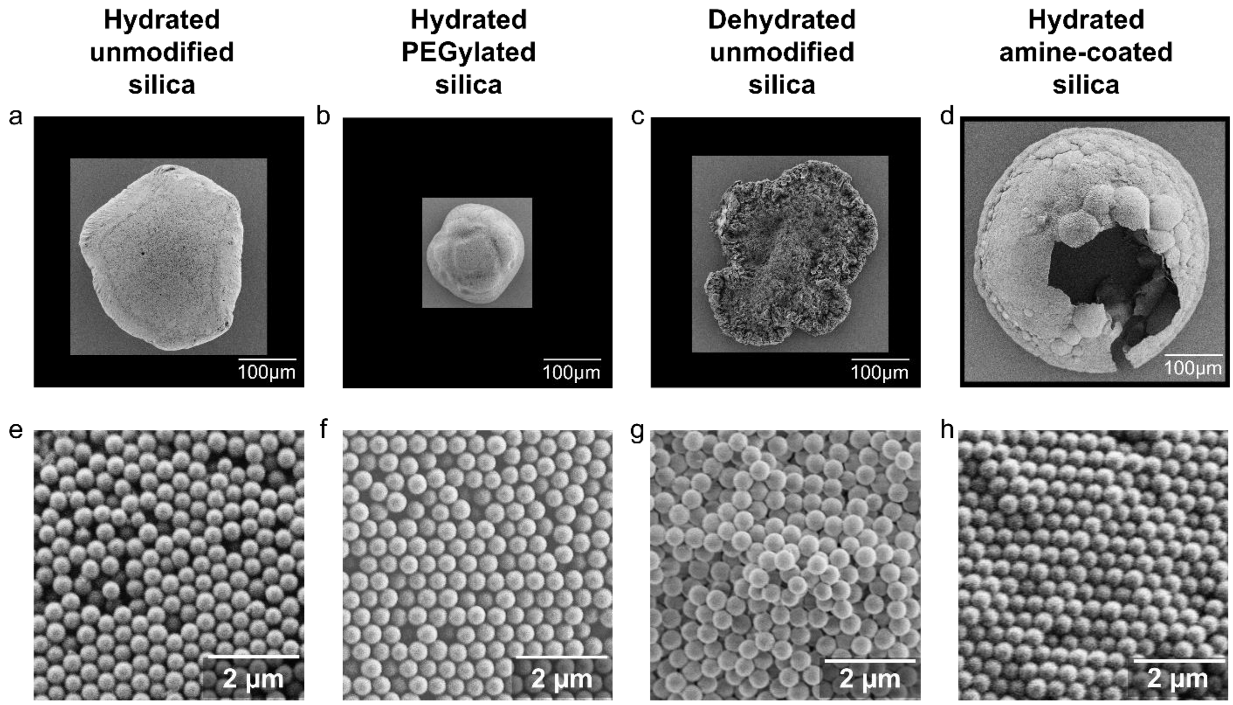

Figure S21. Structure of the final deposits at the surface obtained with different silica particles. (a-d) SEM images showing the top surface of the final deposits. The size of the images are chosen such as to keep same magnification for all images (a) to (d). Scale bar 100 μm. (e-h) Zoomed-in SEM images showing arrangement of the colloidal particles at the surface. Scale bar 2 μm. We note that the height of the deposit of amine-coated particles (d) is similar to the deposit in Figure S3 i.

SEM images show the arrangement of the particles at the surface of the deposits. In the case of hydrated unmodified silica particles, PEGylated silica particles, and amine-coated silica particles (Figure S21 e, f, and h), there are local regions of ordered arrangement of the particles that are separated by disordered regions, leading to relatively smooth surface of the corresponding deposits (Figure S21 a, b, and d). In contrast, the deposit made of unmodified dehydrated particles (c) has a rough/irregular surface (Figure S21 c) because of the largely disordered arrangement of particles (Figure S21 g). The evaporation driven packing of particles in a colloidal droplet into ordered or disordered structures is governed by multiple factors, such as initial particle concentration,[9, 10] drying time,[9] and particle aggregation.[11] In a colloidal Ouzo droplet, there is an additional complexity of particle-oil interactions. Future

studies can address the role of these individual factors in determining the packing of colloidal particles in such multicomponent systems.

S10. References

1. Chevalier, Y.; Bolzinger, M.-A. Emulsions Stabilized with Solid Nanoparticles: Pickering Emulsions. *Colloids Surf., A* **2013,** *439*, 23-34.
2. Binks, B. P.; Lumsdon, S. O. Influence of Particle Wettability on the Type and Stability of Surfactant-Free Emulsions. *Langmuir* **2000,** *16*, 8622-8631.
3. Levine, S.; Bowen, B. D.; Partridge, S. J. Stabilization of Emulsions by Fine Particles I. Partitioning of Particles between Continuous Phase and Oil/Water Interface. *Colloids Surf.* **1989,** *38*, 325-343.
4. Batchelor, G. K. Sedimentation in a Dilute Dispersion of Spheres. *J. Fluid Mech.* **1972,** *52*, 245-268.
5. Tropea, C.; Yarin, A. L.; Foss, J. F., Velocity, Vorticity, and Mach Number. In *Springer Handbook of Experimental Fluid Mechanics*, Springer: Berlin, Heidelberg, 2007; pp 287-289.
6. Diddens, C.; Tan, H.; Lv, P.; Versluis, M.; Kuerten, J. G. M.; Zhang, X.; Lohse, D. Evaporating Pure, Binary and Ternary Droplets: Thermal Effects and Axial Symmetry Breaking. *J. Fluid Mech.* **2017,** *823*, 470-497.
7. Li, Y.; Diddens, C.; Segers, T.; Wijshoff, H.; Versluis, M.; Lohse, D. Evaporating Droplets on Oil-Wetted Surfaces: Suppression of the Coffee-Stain Effect. *Proc. Natl. Acad. Sci. U. S. A.* **2020,** *117*, 16756-16763.
8. Tan, H.; Wooh, S.; Butt, H.-J.; Zhang, X.; Lohse, D. Porous Supraparticle Assembly through Self-Lubricating Evaporating Colloidal Ouzo Drops. *Nat. Commun.* **2019,** *10*, 478.
9. Mukherjee, S.; Saha, A.; Santra, P. K.; Sengupta, S.; Sarma, D. D. Beyond the “Coffee Ring”: Re-Entrant Ordering in an Evaporation-Driven Self-Assembly in a Colloidal Suspension on a Substrate. *J. Phys. Chem. B* **2014,** *118*, 2559-2567.
10. Marín, Á. G.; Gelderblom, H.; Susarrey-Arce, A.; van Houselt, A.; Lefferts, L.; Gardeniers, J. G. E.; Lohse, D.; Snoeijer, J. H. Building Microscopic Soccer Balls with Evaporating Colloidal Fakir Drops. *Proc. Natl. Acad. Sci. U. S. A.* **2012,** *109*, 16455.
11. Sekido, T.; Wooh, S.; Fuchs, R.; Kappl, M.; Nakamura, Y.; Butt, H.-J.; Fujii, S. Controlling the Structure of Supraballs by Ph-Responsive Particle Assembly. *Langmuir* **2017,** *33*, 1995-2002.
